# Cable Monitoring Using Broadband Power Line Communication

**DOI:** 10.3390/s22083019

**Published:** 2022-04-14

**Authors:** Lukas Benesl, Petr Mlynek, Michal Ptacek, Vaclav Vycital, Jiri Misurec, Jan Slacik, Martin Rusz, Petr Musil

**Affiliations:** 1 Department of Telecommunications, Brno University of Technology, Technicka 12, 61600 Brno, Czech Republic; xbenes44@vut.cz (L.B.); misurec@vut.cz (J.M.); xslaci00@vut.cz (J.S.); xruszm00@vut.cz (M.R.); xmusil56@vut.cz (P.M.); 2 Department of Electrical Power Engineering, Brno University of Technology, Technicka 12, 61600 Brno, Czech Republic; ptacekm@vut.cz (M.P.); vycital@vut.cz (V.V.)

**Keywords:** cable diagnostics, smart grid monitoring, health cable monitoring, technical cable coefficient

## Abstract

Power line communication (PLC) is considered one of the possible communication technologies for applications in the field of smart metering, smart substations, smart homes, and recently for the management of renewable resources or micro grid control. This article deals with the use of PLC technology to determine the technical condition of the cable. This coefficient can help distribution system operators (DSO) to assess the condition of their cable routes. In this way, possible cable breakdowns and subsequent power outages can be prevented. The resulting methodology for calculating the coefficient is presented in two specific examples of routes, in which a significant benefit for DSO’s can be found.

## 1. Introduction

Broadband power line communication (BPL) technology is today well known in the commercial sector as power line adapters for home networking [1]. In the industry, BPL was considered in particular for smart meters, but today, thanks to selective roll-out, radio and mobile technologies for point-to-point connection are predominantly considered [2,3].

Nowadays, possible usage of BPL in smart grids is considered mainly for smart secondary substation applications [4,5]. The need to build a smart secondary substation arises from the requirements to more accurately control and monitor the energy system, especially for:

- Power Quality: The distributed power system has been increased with solar and wind power local facilities, which make the grid more heterogeneous and difficult to control.
- Electric Vehicles: Charging and discharging of electric vehicles will be based on available production from solar power generation or other renewable energy sources. Therefore, communication between electric vehicle management, renewable energy sources and data collection supervisory control and data acquisition (SCADA) systems will be necessary.
- Distribution Generation: Implementation of distribution generation requires advanced tools, standards, and guidelines for the secure, reliable and resilient operation of smart grid systems to ensure grid stability, power quality, and cost-effectiveness on the entire value chain of the power network.

BPL is therefore considered for implementation in underground secondary substations, where the signal of mobile networks is not sufficient [4,6].

There are three main advantages of BPL:

- With the growing number of connections of new elements, growing demands on broadband communication and high requirements for cyber security, BPL appears to be a suitable technology that will meet the expected requirements with its parameters. It is also an independent communication network under the administration of utilities (e.g., dependencies on telecommunications operators).
- BPL technology can be deployed directly to existing transformer stations (existing medium voltage (MV) lines), without excavations, and without major intervention. It can be considered for a transitional period and in terms of investment and operating costs, until there is an optical network available everywhere, as the BPL technology is completely sufficient in terms of communication parameters [7].
- Compared to GSM (Groupe Spécial Mobile) and mobile technologies, BPL is a suitable technology for underground transformer stations, where there is no signal and no possibility to pull out an antenna. The most suitable technology would be an optical fiber, but building optical networks is not so simple, especially in city centers. PLC/BPL can show better performance in terms of network-latency, while LTE is proven to be less susceptible to short-term interruptions, resulting in a higher overall reliability [8].

Technology also has disadvantages that many DSOs can discourage from acquiring:

- Communication distance may not be satisfactory. For communication over longer distances, the signal must be amplified or repeated. This brings delays into the whole system and another element that may or may not be necessary for a given route. See also Section 4.1.
- The transmitted signal on the route can attenuate, with a multipath effect [9] where the branches are located, various types of noise [10,11] that can be caused by the power line itself or equipment connected to the network.

Besides the primary use of power line communication (PLC) technology for substation automation and smart metering, the secondary use of PLC could be for cable health monitoring and control in distribution networks.

Cable health monitoring is not a new field of research [12,13,14], but these referenced solutions require dedicated test equipment or manual waveform analysis for data interpretation. Cable health monitoring is an important method in grid monitoring and prevention of grid faults for utilities [15]. Grid faults result in power outages and financial losses, and could also lead to potentially hazardous situations and loss of lives. According to [16,17,18,19,20,21,22,23,24], cable health monitoring and cable fault diagnosis is still an open and challenging research issue in terms of the market and social impact.

The idea used in PLC/BPL modems designated for smart grids communication can also serve as a tool for cable health monitoring, which is a challenging task.

In this paper, we propose a method that exploits the topological and communication parameters to determine the health of distribution cables. The cable health monitoring method based on PLC parameters and cable topological parameters could be used as a utilities diagnostics method for cable renovation planning that can be integrated into SCADA or as a smart grids concept. Monitoring power cables enables utilities to prevent cable failures, which can potentially lead to improving operation reliability and thus improved reliability indexes like the System Average Interruption Duration Index (SAIDI), and the System Average Interruption Frequency Index (SAIFI).

The contribution of this article is threefold. Firstly, the communication testbed for testing and evaluation of communication technologies for the new generation smart substation secondary system was proposed. Secondly, the methodology for verifying the communication parameters of broadband communications considered for the smart substation concept was investigated. Thirdly, the BPL technology was evaluated in a testbed thanks to a universal load generator (traffic generator) which enables the emulation and simulation of the various data flows of smart substations based on IEC 60870-5-104, IEC 61850, and DLMS protocols. The main contribution of the research is the repeatable methodology for evaluation of different communications, standards, or vendors for the smart substation. Based on the evaluation, the recommendation for carrying out BPL technology for the smart substation is proposed.

Besides the existing literature listed in Table 1, our cable diagnostics method uses network topological parameters, cable physical properties, and measured communication parameters of BPL networks.

**Table 1:** State of the art.

No.	Authors	Year	Method	Purpose
[16]	Y. Huo et al.	2018	S ^1^	diagnostic tool for degradation of cables
[17]	G. Prasad et al.	2019	S ^1^	diagnostic tool based on existing PLCs that can measure SNR
[18]	Y. Huo et al.	2018	S ^1^	diagnostic tool for degradation of cables
[19]	A. Poluektov et al.	2018	S ^1^/L ^5^	diagnostic tool for degradation of cables based on BIS, only for LV
[20]	Y. Huo et al.	2019	S ^1^	monitoring cable health conditions based on machine learning framework
[21]	A. Pinomaa et al.	2015	L ^5^	diagnostic tool for degradation of cables based on BIS, only for LV
[22]	Y. Huo et al.	2019	S ^1^	neural networks for cable diagnostics using power line modems
[23]	Y. Huo et al.	2019	S ^1^	automated machine learning based cable diagnostics design
[24]	L. Förstel et al.	2017	S ^1^	PLC as a diagnostic tool for cable aging
[25]	Y. Ohtomo et al.	2010	C ^2^	node detection in topology
[26]	M. Solaz et al.	2014	W ^3^	field and laboratory tests have been run successfully
[27]	C. Freitag et al.	2013	M ^4^	mathematical description of cable degradation without using PLC/BPL
[28]	S. Abeysinghe et al.	2021	A ^6^/S ^1^	modeling of electrical networks in rural, suburban, and urban areas
[29]	A. Siswoyo et al.	2021	S ^1^/E ^7^	simulation and verification by experimental measurements based on BIS
[30]	Y. Kakimoto et al.	2020	S ^1^/E ^7^	partial discharge monitoring system based on HD-PLC communication
[31]	N. Hopfer et al.	2019	L ^5^/F ^8^	analysis of the technical condition of the cable line using BPL
[32]	S. Hu et al.	2018	L ^5^	cable fault diagnosis by SSTDR

^1^ Simulation, ^2^ Concept, ^3^ Working, ^4^ Mathematical, ^5^ Laboratory tests, ^6^ Analysis, ^7^ Experiment, ^8^ Field tests.

Section 2 summarizes the current state of PLC/BPL technology as a diagnostic tool. Section 3 presents work motivation and goals. The theoretical description of individual parameters is contained in Section 4. The proposed methodology and description are part of Section 5. Section 6 contains measurements according to the proposed methodology.

## 2. Related Works—PLC/BPL as a Diagnostic Tool

According to analysis of related works in this Section 2, the method for cable health monitoring is based on topological parameters of the network, physical properties of the cable and measured Quality of Services (QoS) parameters. A similar method of diagnostics is summarized in Table 1, but the works are only based on a mathematical model, which is no longer applied to a specific use case in a real field. Other articles are based on simulations, where a hypothesis is made, and in the end, it is proved by the result of the simulation. Similar works do not consider all three parts of diagnostics (topological parameters of the network, physical properties of the cable, and measured QoS parameters). Thus, this work is different and offers a new perspective on diagnostic solutions.

Publication [16] mentions the use of PLC/BPL technology as a diagnostic tool for detecting cable degradation due to age. The authors examine the theoretical assumption by using simulation, where they create a mathematical model of the cable, including the effects of thermal degradation, and then create a PLC channel. With the help of machine learning and a larger number of samples, they obtain successful results. The authors of article [25] present a method of searching new nodes in already installed PLC/BPL topologies. They use the ALOHA access method for detection, in which they use ACK/NACK messages. It detects a new node by transmission or, conversely, removes it when the node is unavailable, thus determining the status of the line. Article [26] presents several different scenarios that can be used to detect the following types of network errors using BPL technology: backbone fault, BPL device fault, BPL device link fault, coupler fault and connection fault. The authors conducted laboratory and field follow-up tests which proved their assumptions to be successfully verified. Another researcher, Freitag [27], deals with the degradation of paper insulated lead covered (PILC) and cross-linked polyethylene (XLPE) cables depending on the dissipation factor. The author points out that more diagnostic parameters need to be included to increase the accuracy of the diagnostics. Work [17] presents that even older PLC modems can be used to diagnose cable life to prevent malfunctions. The proposed solution is the analysis of available data that the PLC produces and, with the help of machine learning the created algorithm and with a certain probability, can prevent route faults. Simulations in [18] show that water tree (WT) degradation can be detected with an accuracy of more than 90%, assuming a plausible design of channel frequency response (CFR) generation for a specific topology. Simulation design works only for cables with XLPE insulation. Article [19] deals with the use of Broadband Impedance Spectroscopy (BIS) as another possibility of using BPL technology. The cable fault diagnosis method uses the measured channel response and channel gain data, which are further processed. The authors performed a test at the low voltage (LV) level on an AXMK cable with an aluminum core. Fault detection estimation worked relatively accurately. The disadvantage is that the algorithm can only be implemented in BPL modems that use orthogonal frequency division multiplexing (OFDM) modulation. The authors also state that the diagnostic tool can only be used up to 500 m. The model described in article [28] divides the distribution network into two parts, suburban and urban. It takes into account parameters such as area, population density, total number of nodes, total number of branches, number of outgoing feeders from the primary substation, installed capacity of the primary substation, number of secondary substations in the network, total installed capacity of the secondary substation, and maximum feeder length. The resulting model should be used for generation of network statistics. Article [29] deals with the analysis of the localization and detection of degraded parts of a cable using the method BIS. It can be used with both the inverse transformation method and the inverse fast Fourier transform (IFFT) method. Research [30] has focused on partial discharge (PD) monitoring using PLC communication and physical layer (PHY) speed. Temporary changes in PHY speed may indicate PDs. Testing was performed only up to 3 kV voltage. A model based on the use of reflectometry joint time frequency domain reflectometry (JTFDR) using PLC modems to determine the condition of the cable, classification of the cable aging profile, assessment of the severity of cable degradation, and the exact location of the degradation in case of localized degradation, is described in the publication [20]. The authors of [21] performed laboratory testing for a proposed algorithm for cable fault diagnosis, which is designed to detect and locate a location with an accuracy of less than one meter. Testing was performed only on LV cables. The neural network described in article [22] should be used for cable diagnostics. The online monitoring solution uses PLC modems to intelligently diagnose the status of underground power cables through inherently estimated information on the status of the power line communication channel. In [23], the authors propose cable management diagnostics based on machine learning. The performed simulations suggest that the location of cable degradation can be detected with good accuracy only up to 300 m. A diagnostic tool [24] that primarily detects cable aging is involving the WT. The problematic part of the proposed solution was variations caused by cable aging and those due to load changes. Publication [31] shows that attenuation decreases during laboratory testing with cabling degradation. This statement was also confirmed by field testing. Signal to noise ratio (SNR) values in a 15-min interval were used to determine the current state of the cable. Time variation, which may occur due to interference, has also been identified. Article [32] describes the possible detection of cable fault diagnosis by spread spectrum time domain reflectometry (SSTDR), which is a non-intrusive method. The authors test their idea using a simulation, and then experiment on a short cable that is approximately 20 m long. The cable fault was detected within approximately 30 cm of the true fault location.

Dozens of works deal with the use of diagnostics in order to determine the technical conditions of cables. Based on this analysis, the authors of these articles focused on the research of topological properties of underground power line cables with a correlation of QoS parameters on the same route. The list of considered parameters is given in Section 4.

## 3. Motivation and Goals

Prevention and estimation of grid faults is essential for utilities. Grid faults result in power outages and financial losses, and could also lead to potentially hazardous situations and loss of lives.

According to analysis of related works in Section 2, the autonomous method for cable health monitoring based on topological parameters of the network, physical properties of the cable, and measured communication parameters of BPL networks, is missing.

As a part of testing the BPL in a pilot deployment at the distribution company E.ON, problematic power lines were revealed for the BPL communication itself, where communication was not possible at all, but the power line was electrically connected. An electrically interconnected route where BPL communication was not possible at all is described in Section 6.1.

Moreover, other power lines with significantly worse BPL performance and reliability parameters were measured in comparison with similar power lines (BPL network sections). In Section 6.2, a very low transmission throughput for short distances between BPL modems (ideal conditions without branches) is described. The route achieves significantly lower throughput in comparison with other similar routes in a given locality and under the same conditions.

These results led us to further consider what causes inaccessible communication in an electrically connected line, lower throughput or unstable communication (connection failures) for particular lines. Thanks to inaccessible communication and subsequent analyses, problematic sections of lines were discovered, such as wet couplings or poor-quality line couplings, and led and motivated us to create a methodology for using BPL communication outside data transfer as an auxiliary monitoring and diagnostic tool.

The main goals of the article are the following:

- Introduce possible physical properties of underground power line cables, topological parameters of BPL networks and measured communication parameters of BPL networks for a power line cable monitoring and diagnostic method.
- Provide measurements of power line physical parameters and measurements of their influence on BPL performance and power cable life.
- Provide autonomous methodology for cable health monitoring, which could be used by utilities for cable recovery planning.

## 4. Topological Properties of Underground Power Line Cables

The cable’s QoS, or its reliability, can be deduced from various indications. For example, the cable age, its type, and the type of cable joints might be the main driving factors suggesting the cable’s remaining life expectancy. However, as was discussed in Section 2, the PLC/BPL communication technology might also be an early indicator of cable deteriorating conditions. In the following subsections, the power line (and especially the power cable) parameters that might be used for the detection of line deteriorating conditions will be discussed (i.e., short remaining life expectancy and decreased line reliability with higher risk of fault occurrence). The discussed parameters are:

- Distance between BPL modems/cable length,
- cable type,
- cable age,
- cable cross section,
- number of joints on the route,
- joint type installation,
- joint age,
- power loading of the cable,
- partial discharge measurement,
- cable sheath bonding.

These parameters are the main factors that can affect the monitoring of cable health. A detailed explanation of individual parameters is given in Section 4.1–Section 4.11.

### 4.1. Distance between BPL Modems/Cable Length

**Figure 1:**
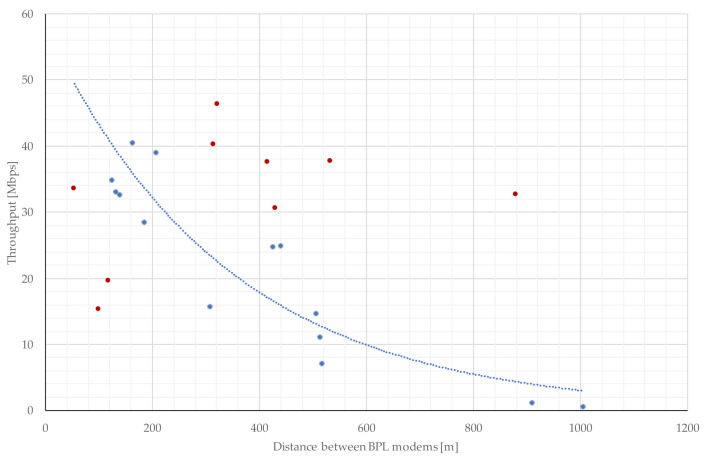
The trend in transmission control protocol (TCP) throughput depending on communication distances between BPL modems on MV lines.

The distance that the BPL signal must travel is one of the most important parameters that will affect the resulting communication. According to simulation and real measurements, BPL communication can be expected in tens of Mbps up to a distance of 600 m [26,33,34] for underground MV power lines. The distances between distribution transformer stations (DTS) are mostly in the range of tens of meters to the higher hundreds of meters for power grids in the Czech Republic. As shown in Figure 1, the field measurements of BPL throughput for point-to-point connection without repeaters has an exponential declining trend for underground MV power lines without branches. In the picture, there are blue dots that are near the curve which, with their predispositions, correspond with the approximate communication throughput concerning the given distance. However, the dots marked in red are too far from the curve, so it is necessary to investigate why. This could be, for example, due to a bad cable, a faulty cable cross section, or a cable joint with pervaded moisture.

The cable length also correlates with the cable failure probability. For example, if the failure probability per unit length of the cable is X, and the cable is n times longer, the likelihood that there will be a failure will be higher due to the unconditional nature of this random process [35]. So, it is very reasonable to include the cable length in the proposed coefficient of the cable condition.

### 4.2. Cable Type

Cables are basic elements of the electrical network. They ensure the interconnection of individual network elements such as transformer stations, electrical sources, and the connection of end customers. High-voltage cables are most often placed in the ground in built-up areas, in most cases to a depth of 80 cm. This is also due to regulations, but it is still possible to meet with overhead power lines. Overhead lines have a wider protection zone for safety reasons, while they have a longer service life and lower construction costs [36]. High voltage cables can be divided into two basic types in terms of the insulation used. The structurally older type of cable is PILC, with paper insulation impregnated with oil. In contrast, newer types are cables with cross-linked polyethylene insulation, referred to as XLPE. XLPE is gradually replacing older PILC cables. At voltage levels of 3 and 6 kV, it is also possible to meet cables insulated with polyvinyl chloride (PVC) or polyethylene (PE), or with cables that have combined insulation PVC with PE.

- PILC cables—on older cable routes, in some cases, high-voltage PILC cables still occur. A tape of cable paper is wound on the surface of the core of the PILC cable, which can reach a thickness of several millimeters to tens of millimeters. After winding, the layer goes through a drying process and then the insulation is impregnated with cable oil. As a result, this oil provides the cable with electrical strength. Impregnating oil together with cellulose paper is the biggest weakness of these cables. Over time, the oil begins to dry, which reduces the electrical strength of the fabric, thus deteriorating the insulating and transmission properties of the cable. Another disadvantage of the PILC cable is its higher weight due to the sheathing of the cable with a lead layer, which results in a more complicated construction of the network [37].
- XLPE cables—cable with cross-linked polyethylene is a variant of linear polyethylene linked polyethylene (LPE). Compared to LPE cables, XLPE cables excel in better mechanical properties at higher thermal loads, usually at operating temperatures up to 90 °C. LPE cross-linking can be achieved by two technologies, the first technology is electron beam irradiation. The second technology is extrusion, in which a layer of LPE is applied and then heated by pressure with added peroxides. This process then results in the required cross-linking [37].

PLC communication will perform better on XLPE cables than on PILC cables. However, it is also possible to use PLC communication for diagnostic purposes on PILC cables [16]. The utility provider has data on cables entered in the Global Information System (GIS), but not everything is always correct. Some records may not be preserved. If BPL technology were used on a cable route, it would be possible to estimate which type of cable it could be according to the throughput speed. In this way, BPL technology can describe the cable and, according to the methodology, also identify the health condition of the cable.

### 4.3. Cable Age

The service life of power lines can fluctuate significantly, mainly due to location (overhead lines/underground lines), as well as due to the geographical characteristics of the environment. The cable life will be different in dry or humid environments, as well as in areas with constant temperature compared to areas with high temperature fluctuations. Overhead lines have an expected service life between 40 and 60 years, while in the case of underground lines, this service life is halved, from 25 to 35 years [38]. The results revealed that the fault current level in the case of an overhead line is significantly smaller than the fault current level in an underground cable.

### 4.4. Cable Cross Section

Power cables for MV are usually produced in a variety of different sizes, i.e., 50, 70, 95, 120, 150, 185, 240, …, 630 mm^2^. Cables with greater cross sections tend to also have a thicker sheath and insulation layers. Therefore, it might take a slightly longer for a water tree defect to evolve into a cable failure [35]. Thus, the cable cross section will be considered for the cable condition coefficient.

### 4.5. Number of Joints on the Route

The power cable failure happens quite often in the cable transition zones, i.e., at the cable ends, or/and at the location of cable joints. Besides both cable ends, there might be a number of cable joints on the cable due to past cable failures, or at installation convenient points (junctions, obstacles etc.). Thus the growing number of junctions on the cable route might increase the cable likelihood to failure. Furthermore, a higher number of cable joints will increase the probability that the BPL communication will not be operational. Based on cooperation with utility providers, there are theoretical assumptions that cable joints have a big influence on the life of the cable line and also on the communication speed. According to real measurements, the BPL communication route was operational with 16 connectors on one cable route. The total length of the cable was 910 m. If it were a single solid cable, the throughput would be higher than the current lower units of Mbps.

### 4.6. Joint Type Installation

There is a large number of cable joint types, but they are divided into two main groups. The first type is the plastic junction and the second type is the heat-shrinkable junction. The process of replacing or installing a junction is a time-consuming operation in which a mistake can be made very easily. If moisture gets into the insulation, the cable will start to break very soon. The problem can be revealed only by a voltage test. As can be seen in Section 6.2, the junction installation type parameter has a significant effect on the resulting communication.

### 4.7. Junction Age

If there is a problem with the cable route, the utility provider must dig up the cable and replace the problematic section with a new cable, connecting the new cable to the old cable using a cable junction. These cable joints are subject to the effects of aging in the same way as cables. Cable joints are prone to moisture ingress, which can shorten the overall life of the cable route. From the point of view of PLC communication, wet cable joints can function as high-frequency filters, which can prevent communication partially or completely. This issue is described in Section 6.1.

### 4.8. Power Loading of The Cable

PLC communication is unstable over time, as different appliances are used at different times, both in households and industrial companies. In cases where there is a large load on the cable route, the capacity of the channel decreases, and thus the quality of the condition of the transmission path deteriorates [39]. A further influence on the load can be caused by the increasing number of branches that can be located on a given route [40].

The different cable loading can also impact the remaining life of the cable because of the varying electrothermal stresses imposed on the cable insulation layers [41]. During the cable nominal operation the cable temperature can increase by several degrees. The generated heat due to resistive and dielectric losses have to be dissipated into the neighboring environment. However, it must be noted that most cables are operated well below their rated loadings and thus they tend to live longer than their designed life.

### 4.9. Correlation of Topological Properties with BPL Communication Parameters

According to analysis of related works in Section 2, the existing methods for cable monitoring or diagnostics are not considering measured communication parameters of BPL networks for correlation with the topological parameters of underground power cables. The BPL throughput and other communication parameters can only be considered where BPL communication is already fully operational. If the communication is operational and some devices are communicating, the throughput, latency, jitter and reliability on the route can be detected or measured in real-time. Thanks to these measurements, these parameters could be used for online autonomous diagnostics. After the installation of BPL, a decrease in average throughput or communication outages can be noted for further analysis. The network administrator can then evaluate that there is something wrong with the route and propose countermeasures. The route can be measured using another diagnostic tool, for example, the very low frequency (VLF) method to prevent possible failure of the cable route. A similar method of evaluation is described in Section 6.

### 4.10. Partial Discharge Measurement

Besides methods taking advantage of new PLC/modem communication monitoring methods, there are still a lot of classical methods for investigating possible QoS of selected lines/cables. Widely adopted methods by the utility companies are the partial discharge and tangent delta measurement. These methods have been proven as a source of information indicating technical condition of power cables.

One of the problems [42] with using these methods is that there is still an ongoing investigation into how to properly read the measurement and extract maximum information about possible improper cable conditions. Thus, the field of correct measurement data interpretation retains a lot of attention and so new assessment frameworks are being proposed [43]. Another problem of the partial discharge measurement is that the measured cable needs to be put out of service and the measurement is usually conducted by the distribution system company employees (i.e., measuring truck, etc.), making this measurement by manpower expensive.

This measurement is therefore conducted quite scarcely, mainly for cables with indices of problems, or as part of routine preventive maintenance. It is quite usual that not every cable condition is assessed by this measurement very often and, for example, in the Czech Republic DSO they are supposed to conduct this measurement only once every four years. Thus, the partial discharge measurement as an early and online indicator of cables deteriorating conditions is not yet very favourable. A possible solution of using this measurement as an early online cable condition indicator might be the adoption/installation of measurement systems such as Smart Cable Guard [44]. The results of partial discharge and tangent delta measurement will be not included in the condition coefficient in the current proposal, however, in case of abundant data availability (like with Smart Cable Guard), it might make the method much more reliable.

### 4.11. Cable Sheath Bonding

High voltage cables are made with sheaths that should increase the protection level in case of failure as well as in case of normal operation. Although most of the concerns with cable sheaths are focused on reducing the level of induced voltage, or the voltage levels during faults, one of the problems with cable sheaths is the presence of circulating currents (CC). The CC usually develop due to the presence of electromagnetic fields (e.g., from other parallel lines) or as potential difference at earthing nodes. The presence of CC is in fact a non-desirable effect because they create additional thermal stress on the cable due to increased power losses, and thus might have a slight effect on cable life expectancy. On the other hand, making the cable sheath grounded on both ends, or even at multiple points, might be beneficial from a PLC/BPL communication point of view, as the cable gets closer to the ideal telegraph cable. From the possible cable bonding/grounding options there are basically four options—no grounding at either end, single end grounding, both ends grounding or multiple points grounding [45] (i.e., respectively no-bonding, single bonding, both end bonding, cross bonding). The optimum case from both a thermal stress and communication interference point of view can be expected for the cross bonding variant. The optimum case for thermal stress alone would be the single end bonding, where no bonding would be impractical and also hazardous, and both end bonding would lead to increased thermal stress. The thermal stress can be further influenced by the fact that some MV cables are still three core cables, or by another factor that is the effect of different cable laying methods.

## 5. Methodology

BPL technology can be considered as an active on-line method of determining the technical health condition of the cable route. It is also possible to use this technology as a diagnostic tool. For this reason, it was possible to design a methodology and create a coefficient for the MV network, which can clearly assess the condition of the cable route.

Each parameter has already been described in Section 4 together with explanation on why it is considered for the methodology. Table 2 indicates what values an individual parameter can take, what the interval of values used in the calculation is (as will be discussed later), and also what the total share of the parameter in the final value of the coefficient is. The parameter of partial discharge measurement is not considered here because the Czech distribution system operator does not have sufficient data that can be used in calculated examples.

Part of the coefficient value is set according to the needs of Czech utility providers. These values can be adjusted as needed by the DSO. The concept of the coefficient is designed so that it can be modified and freely used by others.

The total coefficient starts with number 1 and, by successively subtracting all the 10 sub-coefficients, we get a value in the range 0.000–1.000. Some of the parameters (distance between BPL modems/cable length, number of joints on the route, power loading of the cable, and average TCP throughput) can take values within their intervals. To achieve this, a generic linearization of the actual values to the range interval was used as

(1) $$\mathrm{Coefficient}\;=\;\frac{\mathrm{Actual}\;\mathrm{Value}}{\mathrm{Max}\;\mathrm{Range}}\;\cdot \;\mathrm{Max}\;\mathrm{Coeff}\;\mathrm{Interval}$$

As the dependency in case of cable cross section and TCP throughput are reversed (as lower the actual value as worse the conditions), the formula also needs to be reversed as

(2) $$\mathrm{Coefficient}\;=\;\frac{\mathrm{Max}\;\mathrm{Range}\;-\;\mathrm{Actual}\;\mathrm{Value}}{\mathrm{Max}\;\mathrm{Range}\;-\;\mathrm{Min}\;\mathrm{Range}}\;\cdot \;\mathrm{Max}\;\mathrm{Coeff}\;\mathrm{Interval}$$

For further understanding of using these formulas, see the calculation example in the following Section 5.1. Other parameters are evaluated according to the logical condition: type of cable, whether the junction is heat-shrunk, whether it was installed before 2000, and whether the cable is cross bonded. If the condition is true then the parameter value takes either the upper or lower bound of the interval.

The higher the resulting value of the coefficient, the greater the presumption that the given cable route is in good condition and its early replacement is not expected. If the resulting value of the coefficient is too low, the utility provider should be alerted and first measure the section using VLF methods (tan δ, and partial discharge). This should lead to more frequent voltage tests. If the service life of the cable routes is 50 years, and if we multiply the service life by a coefficient, the result should reduce the service life. If we arrive to a value lower than 5 years, the utility provider should decide, based on both measurements, whether to operate a high-risk cable route or to allocate funds for the renewal of the cable route.

The parameters share on the total coefficient have been chosen so that there is high emphasis on the measured TCP throughput, with this being the leading indicator. The rest of the parameters are more likely connected to the topological properties of the cable, and so the percentage representation of topological and communication influence on the resulting coefficient is set almost identical (45:50). However, it needs to be kept in mind that the PLC/BPL communication is also dependent on some of the topological parameters. The setting of the parameters share has been proposed empirically while taking into account the experience with measurement in the Czech DSOs network and also experience from cited articles of Section 2 and Section 4. It ought to be noted that a more detailed statistical investigation would be beneficial and might be used in future studies to adjust the parameters share of the total coefficient. The current setting of parameters share should avoid the method of falling into deadlock where only long and aging cables would be visited regardless of bad communication performance.

### 5.1. Example of Coefficient Calculation

Thanks to cooperation with the Czech Distribution System Operator (DSO) E.ON, it was possible to perform measurements on a circular topology in the Brno center area. The topology is composed of a total of 26 stations, each station containing a headend and a repeater. The measurement took place between two stations (point-to-point connection). The physical and topological parameters of all measured routes were also provided from the GIS system.

The BPL solution was based on IEEE 1901 OFDM (FFT Access) with a 2–30 MHz frequency band. An example of how to calculate the coefficient for a particular route is given below. Description of the communication route with all available parameters:

**Route A: Substation—DTS1**

Distance between BPL modems: 515.2 m
Cable type: AXEKCY, AXEKCEY, AXEKCEY, AXEKCY, AXEKCEY
Cable age: 1995, 1995, **1979**, **1979**, 1995
Number of cable joints: 4
Cable joint type installation: plastic
Cable joint age:
Load: unknown
Bonding: unknown
Cross section: 240 mm^2^
Average TCP throughput: 8.23 Mbps

(3) $$\mathrm{Route}\;A:\;{\mathrm{Coefficient}}_{\mathrm{Distance}}\;=\;\frac{515.2}{1200}\;\times \;0.05=\;0.0215$$

(4) $$\mathrm{Route}\;A:\;{\mathrm{Coefficient}}_{\mathrm{Cable}\;\mathrm{type}}\;=\;\left(\mathrm{cable}\;\mathrm{not}\;\mathrm{risky}\right)\;=\;0$$

(5) $$\begin{array}{c} \mathrm{Route}\;A:\;{\mathrm{Coefficient}}_{\mathrm{Cable}\;\mathrm{age}}\;=\;\frac{\left(\mathrm{actual}\;\mathrm{year}\;-\;1979\right)}{40}\;\times \;0.15=\;\left(\mathrm{older}\;\mathrm{than}\;40\;\mathrm{years}\right)\;=\;0.1500 \end{array}$$

(6) $$\mathrm{Route}\;A:\;{\mathrm{Coefficient}}_{\mathrm{Number}\;\mathrm{of}\;\mathrm{cable}\;\mathrm{joints}}\;=\;\frac{4}{20}\;\times \;0.15=\;0.0300$$

(7) $$\begin{array}{c} \mathrm{Route}\;A:\;{\mathrm{Coefficient}}_{\mathrm{Cable}\;\mathrm{joint}\;\mathrm{type}}\;=\;\left(\mathrm{plastic}\;\mathrm{not}\;\mathrm{risky},\;\mathrm{but}\;\mathrm{unknown}\right)\;=\;0.0200 \end{array}$$

(8) $$\begin{array}{c} \mathrm{Route}\;A:\;{\mathrm{Coefficient}}_{\mathrm{Cable}\;\mathrm{joint}\;\mathrm{age}}\;=\;\frac{\left(\mathrm{actual}\;\mathrm{year}\;-\;2000\right)}{20}\;\times \;0.15=\;\left(\mathrm{unknown}\right)\;=\;0.1500 \end{array}$$

(9) $$\mathrm{Route}\;A:\;{\mathrm{Coefficient}}_{\mathrm{Load}}\;=\;\frac{\mathrm{load}}{100}\;\times \;0.035=\;\left(\mathrm{unknown}\right)\;=\;0.0350$$

(10) $$\mathrm{Route}\;A:\;{\mathrm{Coefficient}}_{\mathrm{Bonding}}\;=\;\left(\mathrm{unknown}\right)\;=\;0.0100$$

(11) $$\mathrm{Route}\;A:\;{\mathrm{Coefficient}}_{\mathrm{Cross}\;\mathrm{section}}\;=\;\frac{240\;-\;630}{50\;-\;630}\;\times \;0.05=\;0.0336$$

(12) $$\mathrm{Route}\;A:\;{\mathrm{Coefficient}}_{\mathrm{TCP}\;\mathrm{throughput}}\;=\;0.45-\;\frac{8.23\;\times \;0.45}{50}\;=\;0.3750$$

(13) $$\mathrm{Route}\;A:\;{\mathrm{Coefficient}}_{\mathrm{TOTAL}}\;=\;1\;-\;0.826\;=\;0.174$$

Route A shows medium performance, which is mainly influenced by the length of the route itself, which is operated on an old section of cable with a medium number of cable joints. These results correlate with the maximum achieved TCP throughput of slightly over 8 Mbps. The utility provider should regularly monitor this route to see if the maximum throughput is decreasing. Reducing throughput could lead to the detection of a problematic route and prevent cable breakage and subsequent power outages.

**Route B: DTS2—DTS3**

Distance between BPL modems: 118.8 m
Cable type: AXEKCY
Cable age: 1990
Number of cable joints: 0
Junction type installation: without junction
Junction age: without junction
Load: unknown
Bonding: unknown
Cross section: 240 mm^2^
Average TCP throughput: 36.7 Mbps

(14) $$\mathrm{Route}\;B:\;{\mathrm{Coefficient}}_{\mathrm{Distance}}\;=\;\frac{118.8}{1200}\;\times \;0.05=\;0.0050$$

(15) $$\mathrm{Route}\;B:\;{\mathrm{Coefficient}}_{\mathrm{Cable}\;\mathrm{type}}\;=\;\left(\mathrm{cable}\;\mathrm{not}\;\mathrm{risky}\right)\;=\;0$$

(16) $$\mathrm{Route}\;B:\;{\mathrm{Coefficient}}_{\mathrm{Cable}\;\mathrm{age}}\;=\;\frac{\left(\mathrm{actual}\;\mathrm{year}\;-\;1979\right)}{40}\;\times \;0.15=\;0.1163$$

(17) $$\mathrm{Route}\;B:\;{\mathrm{Coefficient}}_{\mathrm{Number}\;\mathrm{of}\;\mathrm{cable}\;\mathrm{joints}}\;=\;\frac{0}{20}\;\times \;0.15=0$$

(18) $$\mathrm{Route}\;B:\;{\mathrm{Coefficient}}_{\mathrm{Cable}\;\mathrm{joint}\;\mathrm{type}}\;=\;\left(\mathrm{no}\;\mathrm{cable}\;\mathrm{joint}\right)\;=\;0$$

(19) $$\mathrm{Route}\;B:\;{\mathrm{Coefficient}}_{\mathrm{Cable}\;\mathrm{joint}\;\mathrm{age}}\;=\;\left(\mathrm{no}\;\mathrm{cable}\;\mathrm{joint}\right)\;=\;0$$

(20) $$\mathrm{Route}\;B:\;{\mathrm{Coefficient}}_{\mathrm{Load}}\;=\;\frac{\mathrm{load}}{100}\;\times \;0.035=\;\left(\mathrm{unknown}\right)\;=\;0.0350$$

(21) $$\mathrm{Route}\;B:\;{\mathrm{Coefficient}}_{\mathrm{Bonding}}\;=\;\left(\mathrm{unknown}\right)\;=\;0.0100$$

(22) $$\mathrm{Route}\;B:\;{\mathrm{Coefficient}}_{\mathrm{Cross}\;\mathrm{section}}\;=\;\frac{240\;-\;630}{50\;-\;630}\;\times \;0.05=\;0.0336$$

(23) $$\mathrm{Route}\;B:\;{\mathrm{Coefficient}}_{\mathrm{TCP}\;\mathrm{throughput}}\;=\;0.45\;-\;\frac{36.7\;\times \;0.45}{50}\;=\;0.1197$$

(24) $$\mathrm{Route}\;B:\;{\mathrm{Coefficient}}_{\mathrm{TOTAL}}\;=\;1\;-\;0.340\;=\;0.660$$

Route B shows almost no wear down. This route is relatively shorter and contains only one undivided cable, so there is no junction on the route. Additionally, the maximum TCP throughput is more than 28 Mbps. The utility provider does not have to supervise this route regularly. The route should be able to operate without any restrictions.

If we compare routes A and B, the coefficient tells us that we need to pay more attention to route A. The cable health is almost half, so the utility provider should monitor the TCP throughput. If the throughput starts to decrease, the route should be measured using the VLF method.

**Interpretation of results:**

Cable life varies with each DSO. Some may be 30 years, others up to 50 years. In our case, we will state, for example, the service life of a cable line at 30 years. If the coefficient for Route A was 0.174, then we multiply this value by the service life of the cables, so it will be 5.22 years. During this time, we should make control measurements using the VLF method at the latest. Route B has a final coefficient of 0.66, if we perform a similar calculation, the final time of further measurement by the VLF method is approximately 20 years. These status messages can help the DSO better alert you to early problem detection before a fault occurs.

## 6. Experimental Measurements of BPL Parameters for Cable Health Monitoring

The VLF method belongs to experimental measurements that are used to determine the service life of a given line. The VLF method uses loss factor measurements tan *δ* (TD) and PD measurements, which are able to determine the state of the cable insulation.

The loss factor TD determines the degree of real power loss in the dielectric material, and thus its losses. The loss factor is more suitable for determining the general condition of the cable insulation, as a whole route. In modeling, the cable insulation system is represented by a simple equivalent circuit consisting of two elements: a resistor and a capacitor. After applying a voltage to the cable, the total current *I* will consist of the capacitor current *IC* and the resistance current *IR*. The loss factor is the ratio of the current of the resistor and the current of the capacitor. the angle TD is the angle between the total current and the charging current when displayed as phasors.

The measurement of partial discharges is closely related to the formation of WT. By measuring PD with source location, it is possible to directly determine the activity of PD in cable sections, connections, and terminals. The passage of the partial discharge pulses depends on the damping in the cable. The measured level, therefore, depends on the distance from the end of the source of PD. Only the time delay between the first and the reflected pulse is important for locating the source of PD. PD inside the cable causes a short-term breakdown of the cable insulation. The pulse-shaped charging current thus generated is detected by means of a coupling capacitor above the measuring device and converted into an equivalent voltage signal. Subsequently, this voltage signal is recorded by the PD detection system and displayed as a pulse on the monitor.

### 6.1. Measurements of Partial Discharges

Experimental measurement with BPL technology was carried out on the distribution territory of a Czech Republic utility provider. A BPL solution was based on IEEE 1901 OFDM (FTT Access) with a 2–30 MHz frequency band. The BPL infrastructure is made up of one substation and 25 stations that are in circular topology. After installing the technology and initial testing, it was found that communication cannot be established between two particular stations. Furthermore, after a closer examination of this route, it was found that the route is electrically connected and operable, although it cannot be communicated using BPL modems. For this reason, a cable measuring car was called that can diagnose the cable. The test includes a voltage test and measurement of partial discharges.

Over time, cables age and their insulation degrades. Thermal overload, humidity ingress, or poorly processed cable joints and cable terminations also contribute to losses. These processes take place over a long period of time. Measuring the loss factor tan *δ* at a frequency of 0.1 Hz makes it possible, as an integral measuring method, to reliably distinguish between new, weak, and heavily aged cables. The measurement of the tan *δ* is an established method for integral diagnostics of the insulation condition on MV cable systems. During the measurement, the absolute limit values are compared with the reference value tan δ. The measurement allows the definition of individual evaluation criteria and creation of a reference database.

**Figure 2:**
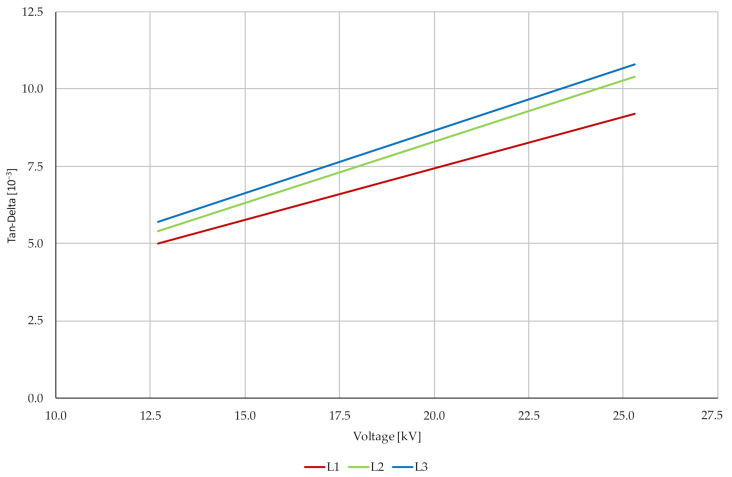
Measurement of tan *δ* using a measuring car.

Figure 2 shows the analysis of TD trends, where the measurement takes place at three voltage levels (12.7, 19.0 and 25.4 kV) and there is a gradual increase in the loss factor on all three phases by almost more than twice the original value. A slight standard deviation is evident between the individual phases. Low PD activity with a mild presence of WT is also indicated.

**Figure 3:**
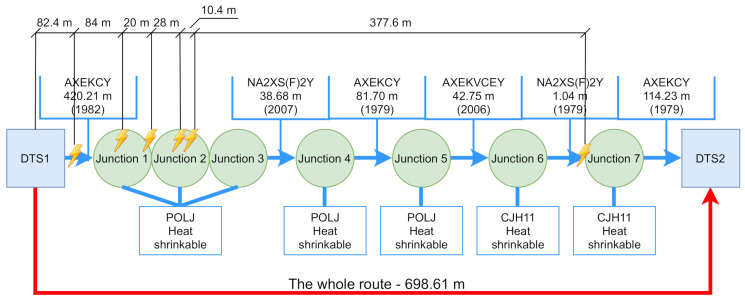
Measurement of the whole route showing cable types, junction types and measurements indicating partial discharges.

Thanks to cooperation with the local DSO, data from GIS were analyzed. Based on the analysis, routes with older cable sections or cable joints were identified, which were then measured using a cable measuring car. Figure 3 shows the entire measured route. The measurement took place from station DTS1 to DTS2. The figure shows a clear description of the topological parameters of the route (cable type, cable age, junction type, junction installation method). In the picture, PDs are visualized using a yellow flash, including the distance from DTS1. These discharges indicate that the cable outer jacket is damaged and the humidity has entered the cable core through the outer jacket. There are also several cable joints on this route. The distance of the discharges from the beginning of the measurement indicates that cable joints 1 and 2 are also affected by humidity. The wet cable joint began to function as a high-frequency filter, making it impossible to communicate using BPL technology. Because of this, part of the cable and the two problematic cable joints are replaced.

After replacement of the problematic cable joints, the subsequent measurements of BPL technology connectivity were made. The results showed the possibility of setting up a connection (communication was enabled), but the connection was not stable. A common type of BPL modems were replaced by the Longhaul type, which operates in the frequency range of 0.75–5 MHz. The disadvantage of Longhaul type BPL modems is low throughput (the measurements show throughput of 7–8 Mbit/s). This set of events revealed another use case of PLC technology. Thanks to the installation of this technology, a problematic section was discovered, which could become inoperable at any time and the customer would be without electricity. A situation where a customer is without electricity is often sanctioned in some countries. Thanks to the early discovery, no customer was disconnected, as the utility provider could prepare in advance for the planned outage and connect the customer using another route.

### 6.2. Measurements of Communication Parameters

Based on the methodology described in Section 5, individual sections of the entire topology of one substation and 25 transformer stations were measured (point-to-point measurements). The goal was to find the bottleneck bandwidth—the lowest value of the bandwidth of the entire measured route [4].

BPL communication can have very unexpected results. The distance between DTS stations is not the only parameter that affects communication. Based on theoretical assumptions [46], throughput decreases with distance. Based on the measurement of the whole topology, two sections were selected for comparison. These sections, named as the longest and the worst, did not correspond to the theoretical assumptions, and the results of the value of the measured throughput also stood out from the remaining sections.

Two routes were compared. Let us call the first route the worst. The route measures 519 m, contains four cable joints and five cables. Let us call the second route the longest. This route measures 880 m, contains five cable joints and six cables. It can therefore be assumed that the worst route will have higher throughput. However, the result is quite the opposite. The communication throughput was approximately six times higher than the worst route in the case of the longest route. The average throughput of the worst route was measured at 5.35 Mbit/s and the longest route reached an average TCP throughput of 32.21 Mbit/s. A comparison of the resulting worst and the longest route communication can be seen in Table 3. The measurement was performed using the EXFO FTB–Pro testers, where the RFC 6349 test methodology was used. The main advantage of the Internet Engineering Task Force (IETF) method RFC 6349 is the fact that it uses the TCP protocol for the measurement itself, which is now predominantly used for non-real-time communication on the internet. This method is standardized for measuring network devices. The size of the TCP window was used by the tester itself, as the most optimal size.

**Figure 4:**
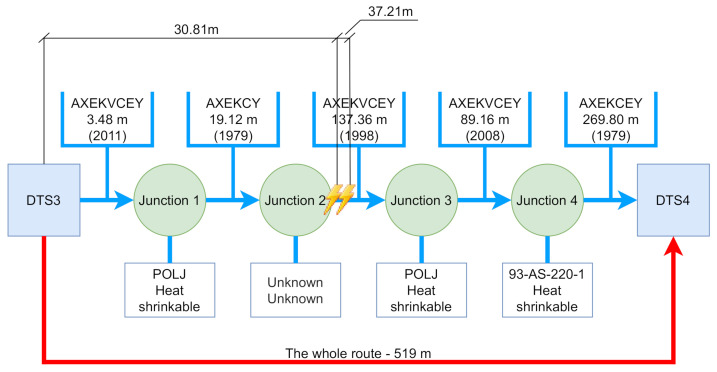
Measurement of the whole **worst route** showing cable types, junction types and measurements indicating partial discharges.

**Figure 5:**
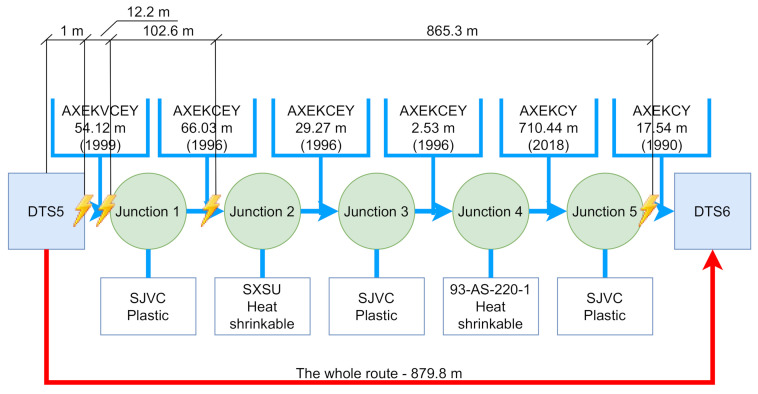
Measurement of the whole **longest route** showing cable types, junction types and measurements indicating partial discharges.

Figure 4 and Figure 5 show the entire measured routes using VLF methods. In both pictures you can see the total length, number and type of cable joints, as well as the lengths and types of individual cable sections. Not all data are always included in GIS data. According to GIS, we know that the junction is located there, but no record of the junction type has been preserved. For this reason, there will be an unknown. The places where the PD’s were detected are marked with a yellow flash, the distance from the starting position is also recorded.

In Table 4, it is possible to clearly compare both measured routes. The table contains an exact description of the topological parameters of the route, which can be found with the GIS tool owned by each utility provider. Data from GIS systems may not always correlate with reality. According to the data, it is not always possible to join two cables with a specific junction. It is therefore necessary to examine the data to see whether they are valid.

A closer look at the table shows that a substantial part of the cable section of the longest route was replaced in 2018. This change of cable had a positive effect on the possible throughput. Although there are several cable joints on the route, they are mostly the plastic junction type that is not as prone to failure and humidity penetration as the heat-shrinkable one. The worst route contains a substantial part of the cable, which was laid in 1979. This cable is then connected to other parts of the cables using heat-shrinkable cable joints.

According to this experience, it can be said very clearly that throughput and QoS parameters also determine the technical condition of the cable.

## 7. Discussion

The use of PLC/BPL modems designed for communication in smart grids as a tool for monitoring the status of the cable is not a completely new idea. Even so, many researchers do not devote themselves to this idea. Our proposed coefficient of the technical condition of the cable route considers a total of 10 parameters that can significantly affect the communication, and thus detect the health of the route itself. The coefficient has a set weighting that corresponds to a certain setting in the Czech distribution network. For the sake of accuracy, it is possible that this weighting will change with an increasing amount of available data, thus increasing the possibility of fault detection.

The data we have so far are only from the MV voltage level. Therefore, this coefficient is focused only for medium voltage networks. Our goal is to expand the coefficient for LV voltage levels and thus increase the possibility of fault detection on more types of lines and make more appropriate use of the already installed BPL technology.

If we compare the current method of underground line diagnostics, it is necessary to find out the main disadvantage. The main disadvantage is that the existing diagnostic tools are mainly offline. This means that for the DSO, it is necessary to plan a shutdown, to secure the supply of energy from other sources, if it is not possible to secure the supply of energy, the affected customers must be informed. In the case of using online diagnostics, it is not necessary to disconnect the measured route, but the measurement/detection of faults takes place constantly. For this reason, the method is more appropriate, not expensive, and if the PLC/BPL infrastructure is available, nothing extra is needed. The method proposed by [21] is based on measuring the broadband impedance response of the power cable. The goal was to create a faster and easier way to monitor cable status. The authors managed to create a method and verify it on AMCMK and AXMK cable types. The cable was broken during testing, so it was an offline method. The proposed algorithm was able to locate the fault within a distance of one meter, but the detection could only be between phases. Our proposed method is independent of the cable type and uses the permeability of the cable joint as one of the main parameters affecting the life of the cable. The proposed method aims to monitor the life of cables, predict when a breakthrough may occur, and prevent this breakdown by the timely and optimal dispatch of a cable measuring car, which used the VLF method for fault localization, loss factor measurement, and partial discharge.

## 8. Conclusions and Future Work

The PLC/BPL technology is in decline due to the roll-out of wireless communications, but where the technology is already installed, it would be a great pity not to use its full potential. The article introduced the secondary use of PLC/BPL communication, especially for cable health monitoring. The article proposed a method for evaluating the condition of cables based on 10 parameters, where each parameter is weighted by its significance. The weighting of the coefficient can be freely adjusted according to the needs of the particular DSO’s. Furthermore, some parameters can be omitted or added according to the needs of the DSO. Thanks to the cooperation with the Czech DSO, the method has been validated in real conditions and the technical condition coefficients of the individual routes were determined. The use of the coefficients can help to optimize the allocation of funds in case of renewal of the power line infrastructure.

The future work will be focused on further measurements in real conditions. This includes working with DSOs who have a PLC/BPL infrastructure. Data collection will be used to refine the coefficient. Obtaining data from cable measurements could help to find a closer connection with the emergence of WT and PD in correlation with the transmission rate. The next step should include verification of the coefficient or adjustment of the weighting of the coefficient. Data should also be collected on transmission parameters of high current cables, such as SNR value, noise, and CFR.

## Figures and Tables

**Table 2:** Rating of individual parameters of the cable health on the MV network.

Parameter	Range of Values	Interval	Part of Coeff. Val.
Distance between BPL modems	1–1200 [m]	0–0.05	5%
Cable type	0 or 1	0 or 0.05	5%
Cable age	0–40 [year(s)]	0 or 0.15	15%
Number of cable joints	0–20	0–0.15	15%
Cable joint type installation	0 or 1	0 or 0.02	2%
Cable joint age	0–40 [year(s)]	0 or 0.05	5%
Load	0–100 [%]	0–0.035	3.5%
Bonding	0 or 1	0 or 0.01	1%
Cross section	50–630 [mm^2^]	0–0.035	3.5%
Average TCP throughput	0–50 [Mbps]	0–0.45	45%

**Table 3:** TCP throughput the worst vs. the longest.

TCP Throughput [Mbps] (TCP Window 43.8 Kbyte)	Worst (519 m)	Longest (880 m)
Average	5.35	32.21
Median	5.29	32.00
Standard deviation	1.35	1.18
Minimum	2.72	28.30
Maximum	8.89	35.70

**Table 4:** Comparison of the worst and the longest measured route.

Worst (519 m)					
	**Cables**		**Cable joints**		
**No.**	**Year**	**Length [m]**	**Type**	**Model**	**Year**
1	2011	3.5			
2	**1979**	19.1	**Heat shrink.**	93-AS-220-1	2011
3	1998	137.4	Heat shrink.	POLJ	2008
4	2008	89.2	Unk.	Unk.	Unk.
5	**1979**	269.8	**Heat shrink.**	POLJ	2008
**Longest (880 m)**					
	**Cables**		**Cable joints**		
**No.**	**Year**	**Length [m]**	**Type**	**Model**	**Year**
1	1999	54.1			
2	1996	66	Heat shrink.	SXSU	1999
3	1996	29.3	Plastic	SJVC	1996
4	1996	2.5	Plastic	SJVC	1996
5	2018	710.4	Plastic	SJVC	1996
6	1990	17.5	Plastic	SJVC	1990

## Data Availability

Author do not will to publish data online.
